# Dose-ranging effects of SGLT2 inhibitors in patients with type 2 diabetes: a systematic review and meta-analysis

**DOI:** 10.20945/2359-3997000000440

**Published:** 2022-01-01

**Authors:** Lana C. Pinto, Dimitris V. Rados, Luciana R. Remonti, Marina V. Viana, Cristiane B. Leitão, Jorge L. Gross

**Affiliations:** 1 Universidade Federal do Rio Grande do Sul Hospital de Clínicas de Porto Alegre Divisão de Endocrinologia RS Brasil Divisão de Endocrinologia, Hospital de Clínicas de Porto Alegre, Programa de Pósgraduação em Ciências Médicas: Endocrinologia, Universidade Federal do Rio Grande do Sul, RS, Brasil

**Keywords:** SGLT2 inhibitors, type 2 diabetes, meta-analysis

## Abstract

The lowest dosage of empagliflozin (10 mg) showed similar benefits on glycated hemoglobin (HbA1c) level, body weight, blood pressure, and total and cardiovascular mortality in comparison with the highest available dose (25 mg) in the EMPAREG trial. These findings have not been clearly demonstrated for canagliflozin and dapagliflozin. The objective was to compare the effect of different doses of SGLT2 inhibitors commercially available in Brazil on HbA1c and body weight of patients with type 2 diabetes. MEDLINE, Cochrane and Embase databases were searched from inception until 11^th^ October 2021 for randomized controlled trials of SGLT2 inhibitors in type 2 diabetes patients, lasting at least 12 weeks. HbA_1c_ and body weight variations were described using standard mean difference. We performed direct and indirect meta-analysis, as well as a meta-regression with medication doses as covariates. Eighteen studies were included, comprising 16,095 patients. In the direct meta-analysis, SGLT2 inhibitors reduced HbA_1c_ by 0.62% (95% CI −0.66 to −0.59) and body weight by 0.60 kg (95% CI −0.64 to −0.55). In the indirect meta-analysis, canagliflozin 300 mg ranked the highest regarding reductions in HbA_1c_ and body weight. The remaining medications and dosages were clinically similar, despite some statistically significant differences among them. Canagliflozin 300 mg seems to be more potent in reducing HbA1c and body weight in patients with type 2 diabetes. The remaining SGLT2 inhibitors at different doses lead to similar effects for both outcomes. Whether these glycemic and weight effects are reflected in lower mortality and cardiovascular events is still uncertain and may be a topic for further studies.

## INTRODUCTION

Sodium-glucose cotransporter 2 (SGLT2) inhibitors are a class of antihyperglycemic medications that inhibit renal glucose reabsorption in the proximal convoluted renal tubule and lead to glucosuria (1-3). SGLT2 inhibitors also have a beneficial effect on blood pressure (BP) and body weight (4-6).

Canagliflozin, dapagliflozin and empagliflozin are the three SGLT2 inhibitors currently approved by the Food and Drug Administration (FDA) for clinical use and the usual recommended doses are 300 mg, 10 mg and 25 mg, respectively (7). However, in the EMPA-REG Outcome trial, a smaller dose of empagliflozin (10 mg) produced similar benefits on glycated hemoglobin (HbA_1c_) level, body weight and blood pressure in comparison with the highest available dose (25 mg) (8). Most importantly, the reduction in total and cardiovascular mortality was comparable for both doses (8), suggesting no dose-dependent effect for any of the evaluated outcomes. Data regarding canagliflozin and dapagliflozin at different doses are lacking, since the Canvas Trial failed to find separate results for both doses of canagliflozin and Declare TIMI 58 only used dapagliflozin 10 mg as an experimental group (9,10). As there are no head-to-head studies comparing the different SGLT2 inhibitors, it is uncertain if the other two agents, canagliflozin and dapagliflozin, behave similar to empagliflozin.

Thus, the aim of this study was to analyze the efficacy of different SGLT2 inhibitor doses compared to placebo and each other in patients with type 2 diabetes regarding HbA_1c_, body weight and adverse events.

## METHODS

### Protocol and registration

This systematic review and meta-analysis follows the recommendations of the PRISMA (Preferred Reporting Items for Systematic Reviews and Meta-analyses) protocol (11) and is part of the project registered at PROSPERO (the International Prospective Register of Systematic Reviews) (CRD42015006975).

### Information sources and search strategy

We performed a systematic literature search for randomized clinical trials (RCTs) that compared SGLT2 inhibitors commercially available in Brazil with placebo. We searched MEDLINE, Embase, Cochrane Central and Clinicaltrials.gov from database inception to January 2018 and abstracts published in the most recent American Diabetes Association and the European Association for the Study of Diabetes meetings. We also performed two search updates in November 2020 and 11^th^ October 2021, meaning that all published papers until last search were screened and included if appropriate. The search strategy combined the Medical Subject Heading (MeSH) terms “dapagliflozin” OR “canagliflozin” OR “empagliflozin” AND “diabetes mellitus, type 2” AND a validated filter to identify RCTs (12). All eligible trials were considered for review, regardless of language. Manual search of reference lists of key articles was also performed.

### Eligibility criteria

The inclusion criteria were: (I) RCTs, (II) SGLT2 inhibitors as experimental treatment, (III) treatment duration for a minimum of 12 weeks, (IV) description of variation in HbA_1c_ or body weight, and (V) inclusion of adult patients (≥18 y old) with type 2 diabetes (13).

### Study selection and data collection

Two independent investigators (L.C.P. and D.V.R.) selected studies based on titles and abstracts. Studies satisfying the inclusion criteria or those with abstracts that lacked crucial information to decide upon their exclusion were retrieved for full-text evaluation. Both investigators (L.C.P. and D.V.R.) also analyzed the trials selected for detailed analysis and extracted data, and disagreements were resolved by consensus. We extracted the following information: first author's name, year of trial publication, sample size and dropouts, age, gender, mean diabetes duration, trial duration, treatment used prior to randomization, doses of SGLT2 inhibitors, change in HbA_1c_ (mean [SD]), change in body weight in kilograms (mean [SD]) and adverse events: hypoglycemia (any event), bone fractures (any fracture), urinary tract infection and genital mycotic infection.

### Risk of bias in individual studies and the quality of meta-analysis

The quality of the studies was assessed according to the Cochrane Collaboration tool for risk of bias (14,15). The quality of each meta-analysis was evaluated using the Grading of Recommendations Assessment, Development and Evaluation (GRADE) approach (16), considering factors that may decrease or increase the quality of evidence. As recommended, each meta-analysis was rated as high, moderate, low or very low quality (16).

### Synthesis of results

First, we analyzed different SGLT2 inhibitor doses versus placebo. The outcomes of interest were absolute changes in HbA_1c_, body weight and adverse events. Continuous variables were expressed as standard mean differences and 95% confidence interval (CI). Discrete events (urinary tract infections, genital infections, hypoglycemia) were expressed as relative risk (RR) and 95% CI. Direct meta-analyses were used to compare individual SGLT2 inhibitor doses with placebo. A separate indirect meta-analysis was conducted for both change in HbA_1c_ and body weight to compare the different doses with placebo, as well as with each other. The Cochran Q test was used to evaluate heterogeneity among studies, and a threshold *p*-value of 0.1 was considered statistically significant; the *I*^2^ test was also conducted to evaluate the magnitude of the heterogeneity among studies.

If heterogeneity in the meta-analysis was high (I^2^ > 75%), we planned to use meta-regression to assess the variables involved in this heterogeneity. We assessed the possibility of small-study bias using a funnel plot of each trial's effect size against the standard error. Funnel plot asymmetry was also evaluated using Begg's and Egger's tests, and a bias was considered to be present if the *p*-value was < 0.1 The trim-and-fill computation was used to estimate whether the unpublished would influence the interpretation of results (17,18).

The analyses were performed using Stata version 12.0 (Stata Inc., College Station, Texas, USA). Indirect meta-analyses were performed using R version 3.4.3 (R Foundation for Statistical Computing, Vienna, Austria). Risk of bias was analyzed using RevMan software version 5.3 (Cochrane Collaboration, Copenhagen, Denmark).

## RESULTS

### Literature search

Our search retrieved 1,556 articles. After removal of duplicated papers and scanning titles and abstracts, 103 articles remained for whole-text evaluation. Subsequently, 18 RCTs were included for analysis (Figure 1S).

### Study characteristics and risk of bias

The included trials were published from 2009 to 2018. These analyses included 16,095 patients, of whom 10,043 were men (62.39%). Detailed study characteristics are shown in Table 1.

**Table 1:** Characteristics of the included trials

Author Year	n	Follow-up (wk)	Men (%)	Mean age (y)	Mean diabetes duration (y)	Mean HbA_1c_ (%)	Mean weight (kg)	Background treatment	SGLT2 inhibitor/dose
Bailey 2013 (26)	546	24	53.48	59.9	NR	8.05	85.91	Metformin	Dapagliflozin 2.5 mg Dapagliflozin 5 mg Dapagliflozin 10 mg
Wilding 2012 (25)	808	48	47.28	59.3	13.6	8.53	93.82	Insulin and/or OAD	Dapagliflozin 2.5 mg Dapagliflozin 5 mg Dapagliflozin 10 mg
Wilding 2009 (27)	71	12	59.15	56.7	12.3	8.43	102.10	Insulin	Dapagliflozin 10 mg Dapagliflozin 20 mg
Rosenstock 2012 (28)	420	48	49.52	53.4	NR	8.37	86.30	Pioglitazone	Dapagliflozin 5 mg Dapagliflozin 10 mg
Bode 2013 (29)	714	102	55.46	52.9	11.7	7.70	89.50	Naïve or OAD	Canagliflozin 100 mg Canagliflozin 300 mg
Wilding 2013 (30)	343	12	51.02	57.4	5.9	7.76	89.76	Metformin	Canagliflozin 100 mg Canagliflozin 300 mg
Zinman 2015 (8)	7020	192	71.45	63.1	NR	8.07	86.3	Any	Empagliflozin 10 mg Empagliflozin 25 mg
Yang 2018 (31)	275	24	47.8	57.5	12.5	8.55	71.8	Insulin ± OAD	Dapagliflozin 10 mg
Januzzi 2017 (32)	714	26	55.4	63.6	NR	NR	NR	OAD	Canagliflozin 100 mg Canagliflozin 300 mg
Haering 2015 (33)	666	76	50.9	57.1	NR	8.1	76.9	Metformin + sulphonylurea	Empagliflozin 10 mg Empagliflozin 25 mg
Jabbour 2014 (34)	447	24	54.80	54.8	5.67	7.95	90.1	Sitagliptin ± metformin	Dapagliflozin 10 mg
Matthaei 2015 (35)	216	24	49.07	61	9.4	8.16	89.35	Metformin + sulphnylurea	Dapagliflozin 10 mg
Leiter 2014 (36)	964	24	67.01	63.7	NR	8.0	93.8	OAD	Dapagliflozin 10 mg
Stenlöf 2013 (37)	584	26	44.18	55.4	4.3	8.00	86.8	Diet + exercise	Canagliflozin 100 mg Canagliflozin 300 mg
Roden 2013 (38)	899	24	61	55	NR	7.88	78.4	No treatment for at least 12 weeks	Empagliflozin 10 mg Empagliflozin 25 mg
Tikkanen 2015 (39)	823	12	60.1	60.2	NR	7.90	NR	Diet + exercise	Empagliflozin 10 mg Empagliflozin 25 mg
Yale 2014 (40)	269	52	60.59	68.5	16.3	8.00	NR	Insulin	Canagliflozin 100 mg Canagliflozin 300 mg
Fulcher 2016 (41)	316	18	65.50	63.0	12.6	8.1	NR	DPP-4 inhibitor	Canagliflozin 100 mg Canagliflozin 300 mg

The comparator for all trials is placebo. Abbreviation: wk = week; y = years; OAD = oral antidiabetic dose

Details regarding the assessment of risk of bias for individual studies and across studies are presented in the additional material (Figure 2S). Random sequence generation, allocation concealment and blinding of outcome assessment were clear in most studies; blinding of participants and personnel, incomplete outcome data and selective reporting were considered to have a low chance of bias in most studies.

### Main outcomes

The overall reduction in HbA_1c_ was 0.62% (95% CI −0.66% to −0.59%; *I*^2^ 92%) when all medications and dosages were analyzed together. In direct meta-analysis, canagliflozin 300 mg produced the greatest numerical reduction in HbA_1c_ (-0.79%; 95% CI: −0.84% to −0.75%; *I*^2^ 97%), whilst dapagliflozin 2.5 mg resulted in the smallest reduction (-0.35%; 95% CI −0.45% to −0.26%; *I*^2^ 0%) (Figure 1). Regarding body weight, canagliflozin 300 mg also had the greatest reduction in body weight (-2.36 kg; 95% CI −2.74 kg to −1.98 kg; *I*^2^ 76%) and dapagliflozin 2.5 mg had the smallest benefit (-1.31 kg; 95% CI −1.78 kg to −0.84 kg; *I*^2^ 71.6%) (Figure 1). The results of indirect and network meta-analysis are similar to those of the direct meta-analysis: in terms of HbA_1c_ reduction, canagliflozin 300 mg was superior to all other SGLT2 inhibitors at different doses, dapagliflozin 10 mg was similar to empagliflozin 10 mg, but inferior to empagliflozin 25 mg, and both doses of empagliflozin (10 mg and 25 mg) were similar to canagliflozin 100 mg.

**Figure 1:**
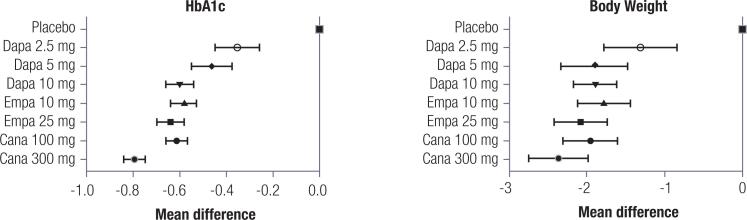
Mean difference in HbA1c and body weight according to SLGT2 inhibitor and dose.

Regarding body weight, canagliflozin 300 mgalso had the greatest benefit in terms of body weight reduction; however, it was not different from empagliflozin 25 mg and dapagliflozin 10 mg. The results of both direct (against placebo) and indirect meta-analyses are shown in Table 2.

**Table 2:** Network meta-analysis for each SGLT2 inhibitor dose regarding the effects on HbA1c (grey) and body weight (white)

Dapa 2.5	0.11 [-0.001; 0.22]	0.25 [0.14; 0.35]	-0.35 [-0.44; −0.25]	0.23 [0.12; 0.34]	0.29 [0.18; 0.40]	0.26 [0.16; 0.37]	0.44 [0.33; 0.54]
0.58 [0.02; 1.14]	**Dapa 5**	0.13 [0.04; 0.23]	-0.46 [-0.55; −0.38]	0.12 [0.02; 0.22]	0.18 [0.07; 0.28]	0.15 [0.05; 0.25]	0.33 [0.23; 0.42]
0.57 [0.06; 1.07]	-0.01 [-0.46; 0.44]	**Dapa 10**	-0.59 [-0.66; −0.54]	-0.01 [-0.09; 0.07]	0.04 [0.04; 0.12]	0.01 [-0.06; 0.09]	0.19 [0.12; 0.27]
-1.31 [-1.78; −0.84]	-1.89 [-2.32; −1.47]	-1.88 [-2.16; −1.60]	**Placebo**	0.58 [0.64; 0.52]	0.64 [0.69; 0.58]	0.61 [0.57; 0.66]	0.79 [0.75; 0.84]
0.46 [-0.11; 1.04]	-0.11 [-0.65; 0.42]	-0.10 [-0.54; 0.33]	1.77 [1.44; 2.11]	**Empa 10**	-0.05 [-0.00; 0.10]	0.03 [-0.10; 0.04]	0.21 [0.13; 0.28]
0.76 [0.18; 1.34]	0.18 [-0.35; 0.71]	0.19 [-0.24; 0.62]	2.07 [1.74; 2.41]	0.29 [0.01; 0.58]	**Empa 25**	-0.02 [-0.04; 0.09]	0.15 [0.08; 0.22]
0.64 [0.05; 1.23]	0.05 [-0.49; 0.60]	0.07 [-0.38; 0.52]	1.95 [1.60; 2.30]	0.17 [-0.31; 0.66]	-0.12 [-0.60; 0.36]	**Cana 100**	0.18 [0.13; 0.22]
1.04 [0.43; 1.65]	0.46 [-0.11; 1.03]	0.47 [0.001; 0.94]	2.35 [1.97; 2.73]	0.58 [0.07; 1.08]	0.28 [-0.22; 0.78]	0.40 [0.01; 0.79]	**Cana 300**

**Figure 3S:**
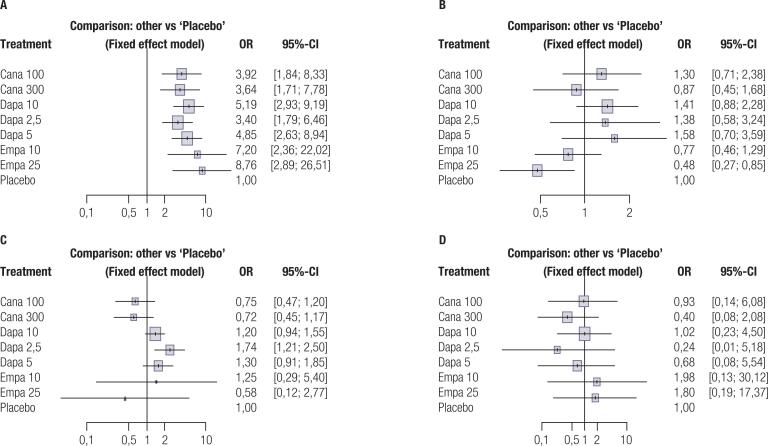
Adverse events related to SGLT2 inhibitors use. **A**. Genital mycotic infection; **B**. Urinary tract infection; **C**. Hypoglycemia; **D**. Bone fracture.

None of the included trials showed a difference in incidence of adverse events when using different doses of SGLT2 inhibitors, so it was not possible to analyze this outcome by dosage. None of the SGLT2 inhibitors, at any of the studied doses, increased risk of urinary tract infection or bone fractures. Only dapagliflozin 2.5 mg increased the risk of hypoglycemia. All SGLT2 inhibitors at different doses were associated with increased risk of genital mycotic infection (Supplemental Material – Figure 3S).

As heterogeneity among trials was high, we performed a meta-regression using medication dose as a covariate, which did not explain the heterogeneity found.

### Meta-analysis quality evaluation

The GRADE quality of evidence was considered high, but was downgraded one point due to indirectness. No publication bias was identified in the meta-analysis (p = 0.441).

## DISCUSSION

The present study shows that SGLT2 inhibitors have similar effects on HbA_1c_ and body weight regardless of the agent used and the employed dosage. Some minor differences were found in the indirect analysis of canagliflozin 300 mg; however, the clinical significance of this difference (0.2% in HbA1c and less than 500 g in body weight) is questionable. Regarding adverse events, all SGLT2 inhibitors at different doses were associated with genital mycotic infections, but not with bone fractures or urinary tract infection.

Other meta-analyses showed similar findings to ours regarding the effects of SGLT2i on HbA1c (19,20). Both of these analyses included trials that lasted more than 24 weeks and one also analyzed the efficacy of SGLT2 inhibitors compared with other agents (19). However, these previous studies did not explore the effects of different doses of SGLT2 inhibitors, nor did they compare their effectiveness compared to each other. Our results are in accordance with a large trial of an SGLT2 inhibitor, the EMPAREG Outcomes trial (8), where the two tested doses of empagliflozin had the same effect on cardiac outcomes, body weight and HbA_1c_. Another published cardiovascular trial of SGLT-2 inhibitors, the CANVAS trial, did not show the results for canagliflozin 100 mg and 300 mg separately, so their results were not included in this analysis (9). Moreover, more recent studies, such as the Declare TIMI 58 trial, also did not present extractable results of glycemic control in randomized patients (10), and neither did the trials that randomized patients with heart failure to SGLT-2 inhibitors, DAPA-HF, Emperor Reduced and Emperor Preserved Trials (21-23). Nonetheless, we must emphasize that in the latter two trials, cardiovascular benefits were seen with the lowest dose of empagliflozin, as patients were only randomized for 10 mg of empaglifozin (22,23).

The finding of greater reduction in HbA_1c_ and body weight with canagliflozin 300 mg should be interpreted carefully. This reduction is expected since canagliflozin is the least selective among the three SGLT2 inhibitors, also leading to SGLT1 inhibition in the distal part of the convoluted proximal tubule (S3 segment) and intestine (24). This particular characteristic may increase the level of glucosuria or decrease intestinal absorption of glucose. However, it is important to highlight that the greater benefits found with canagliflozin 300 mg, even though statistically significant in relation to other medications/doses, may not be clinically relevant, as they represent a reduction of approximately 0.2% in HbA1c and less than 500 g in body weight. Therefore, the differences reported herein should not be taken into consideration when choosing a particular SGLT2 inhibitor. The findings of the CANVAS trial should also be taken into account, while the greater incidence of amputations in patients randomized to canagliflozin remains unexplained (9).

There was a small increase in risk of hypoglycemia with dapagliflozin 2.5 mg that could be related to the studies included, which randomized patients on high doses of insulin to SGLT2 inhibitors (25).

We must emphasize some of the strengths of these results: we performed a thorough search of the databases, the findings were consistent across the outcomes and the quality of primary studies was high. As heterogeneity between the included trials was high, we also performed a meta-regression using the studied doses of canagliflozin, dapagliflozin and empagliflozin as covariates, which did not explain the heterogeneity found, increasing our confidence in the results.

Our results have practical and economic implications. It is not worthwhile to increase SGLT2 inhibitor doses with the intent to further decrease HbA_1c_ or body weight. Further, in the light of our results, we believe that these medications should be produced in a single dosage formulation.

Unfortunately, some additional information was lacking in the majority of the studies and we were therefore unable to explore some interesting additional topics, such as blood pressure reduction, side effects, mortality, and cardiovascular events.

In conclusion, the current review shows that the lowest commercially available doses of SGLT2 inhibitors have similar clinical effects on HbA1c and body weight to the higher doses. More evidence is needed to elucidate the effects of different doses on blood pressure, major cardiovascular events and death. Whether these glycemic and weight effects are reflected in mortality and cardiovascular events is still uncertain and may be a topic for further studies.

## Figures and Tables

**Figure 1S:**
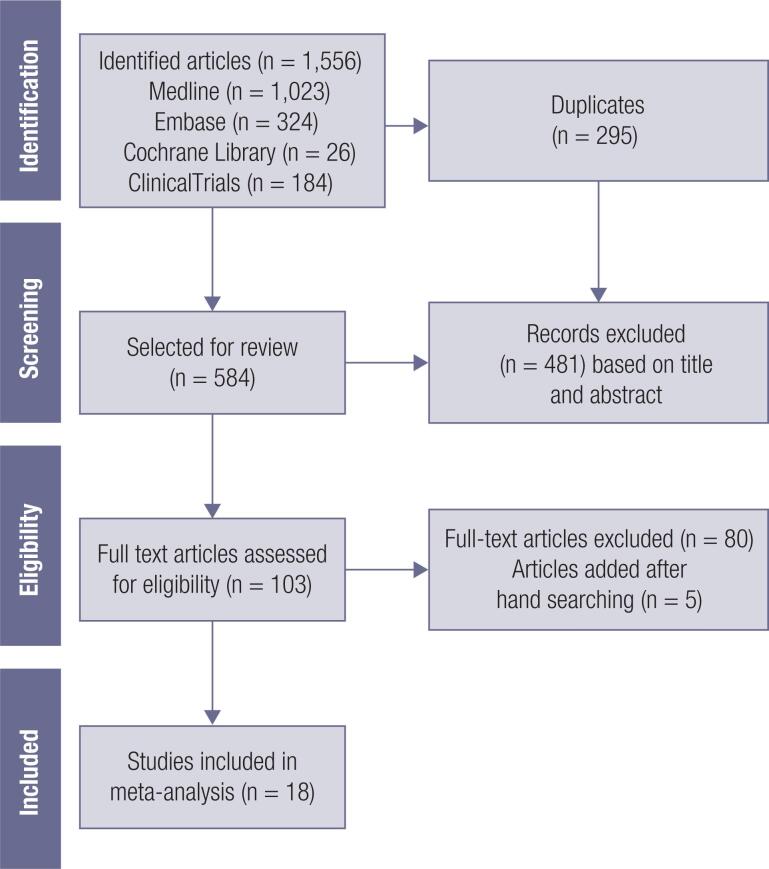
Study flowchart.

**Figure 2S:**
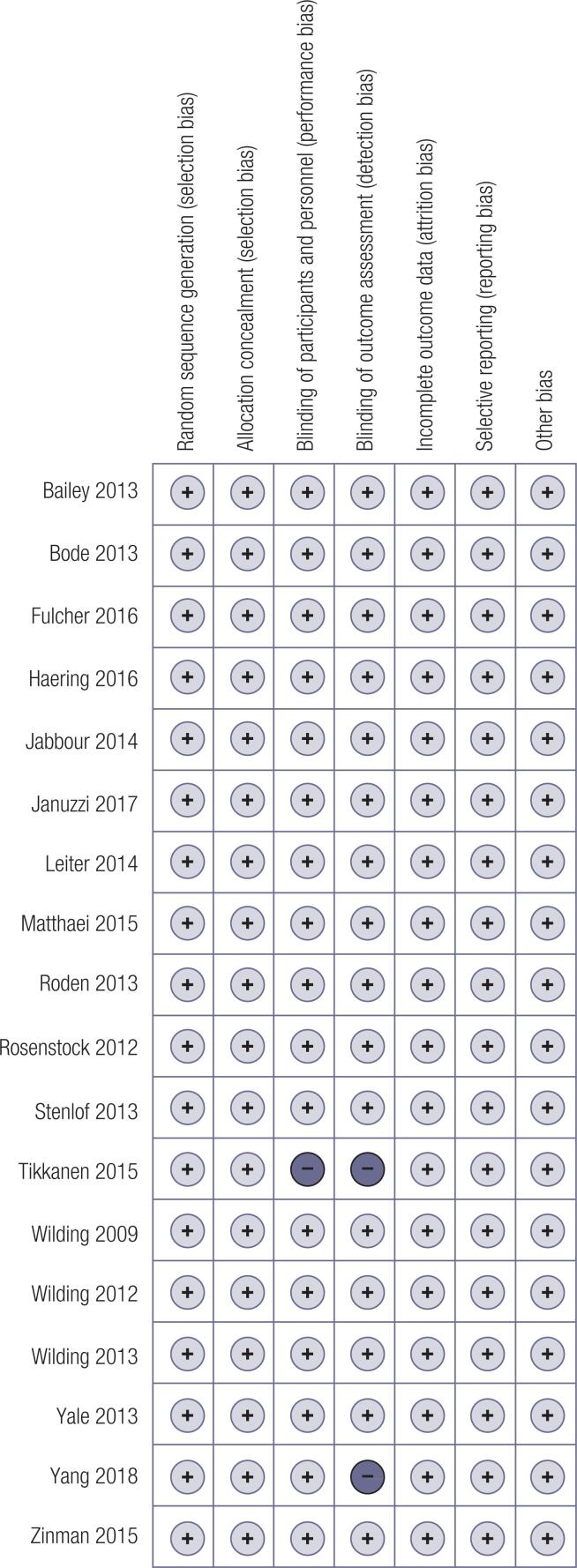
Risk of bias
